# Histidine-Rich Glycoprotein Uptake and Turnover Is Mediated by Mononuclear Phagocytes

**DOI:** 10.1371/journal.pone.0107483

**Published:** 2014-09-22

**Authors:** Sònia Tugues, Francis Roche, Oriol Noguer, Anna Orlova, Sujata Bhoi, Narendra Padhan, Peter Åkerud, Satoshi Honjo, Ram Kumar Selvaraju, Massimiliano Mazzone, Vladimir Tolmachev, Lena Claesson-Welsh

**Affiliations:** 1 Department of Immunology, Genetics and Pathology, The Rudbeck Laboratory, Uppsala University, Uppsala, Sweden; 2 Department of Medicinal Chemistry, Preclinical PET Platform, Uppsala University, Uppsala, Sweden; 3 Department of Surgical Sciences, Uppsala University, University Hospital, Uppsala, Sweden; 4 Laboratory of Molecular Oncology and Angiogenesis, Vesalius Research Center, Dept of Oncology, Katholieke Universiteit Leuven, Leuven, Belgium; 5 Laboratory of Molecular Oncology and Angiogenesis, Vesalius Research Center, Vlaams Instituut voor Biotechnologie, Leuven, Belgium; 6 Department of Radiology, Oncology and Radiation sciences, The Rudbeck Laboratory, Uppsala University, Uppsala, Sweden; University of Bristol, Bristol, UK, United Kingdom

## Abstract

Histidine-rich glycoprotein (HRG) is implicated in tumor growth and metastasis by regulation of angiogenesis and inflammation. HRG is produced by hepatocytes and carried to tissues via the circulation. We hypothesized that HRG's tissue distribution and turnover may be mediated by inflammatory cells. Biodistribution parameters were analyzed by injection of radiolabeled, bioactive HRG in the circulation of healthy and tumor-bearing mice. ^125^I-HRG was cleared rapidly from the blood and taken up in tissues of healthy and tumor-bearing mice, followed by degradation, to an increased extent in the tumor-bearing mice. Steady state levels of HRG in the circulation were unaffected by the tumor disease both in murine tumor models and in colorectal cancer (CRC) patients. Importantly, stromal pools of HRG, detected in human CRC microarrays, were associated with inflammatory cells. In agreement, microautoradiography identified ^125^I-HRG in blood vessels and on CD45-positive leukocytes in mouse tissues. Moreover, radiolabeled HRG bound in a specific, heparan sulfate-independent manner, to differentiated human monocytic U937 cells *in vitro*. Suppression of monocyte differentiation by systemic treatment of mice with anti-colony stimulating factor-1 neutralizing antibodies led to reduced blood clearance of radiolabeled HRG and to accumulation of endogenous HRG in the blood. Combined, our data show that mononuclear phagocytes have specific binding sites for HRG and that these cells are essential for uptake of HRG from blood and distribution of HRG in tissues. Thereby, we confirm and extend our previous report that inflammatory cells mediate the effect of HRG on tumor growth and metastatic spread.

## Introduction

The heparin-binding protein histidine-rich glycoprotein (HRG) has received increasing attention due to its role in diverse processes such as bacterial infection and tumor development [1], [2]. Structurally, the 506 amino acid HRG polypeptide consists of two N-terminal cystatin-homology domains, classifying HRG as a member of the soluble (class 3) subfamily of cysteine protease inhibitors together with fetuin A, fetuin B and kininogen [3], [4]. The cystatin domains are followed by a stretch of 12 penta-peptide repeats with the consensus sequence HHPHG, denoted the His-Pro domain. HRG's biological activity is dependent on binding of Zn^2+^ to the His-Pro domain [5]. The histidine residues in the His-Pro domain as well as the N-terminus of HRG bind heparan sulfate (HS) [6], [7]. The three-dimensional structure of HRG has not been solved, but the presence of several intramolecular disulfide bridges [8] indicates that native HRG attains a tight, globular conformation.

HRG is produced exclusively in liver hepatocytes [9] and is distributed systemically both unbound and in platelets. It is found in plasma at relatively high concentrations of 100–150 µg/ml in healthy adults. The HRG concentration in plasma increases with age but decreases in diseases such as advanced liver cirrhosis, AIDS, renal disease and pulmonary disorders [10], [11], [12]. Gene knockout of *hrg* does not interfere with embryonic development, but is accompanied by increased clot formation as well as increased fibrinolysis [13]. There are a few cases of familial HRG mutations that result in reduced plasma HRG levels without a direct correlation with thrombotic events [7]. A potential hemostatic role of HRG could mechanistically be due to its interaction with both fibrinogen and thrombospondin [13].

HRG appears to have a major role in the modulation of inflammatory reactions including the regulation of Fcγ receptor expression and phagocytosis [14]. Moreover, HRG is essential in mounting inflammatory and immune responses against bacterial and fungal infections [2], [15]. In cancer, HRG polarizes tumor-associated macrophages from a pro-angiogenic, immune-suppressive M2 phenotype towards an anti-tumor, immunity-promoting, M1 phenotype [1], [16]. It has been suggested that HRG's bioactivity correlates with fragmentation of the protein [17], [18].

In the present study, we show for the first time that mononuclear phagocytes, primarily consisting of monocytes and macrophages, present specific binding sites for HRG and that these cells are critical in HRG's biodistribution and turnover. Thereby, we provide information essential in further development of HRG-based therapeutics for diseases characterized by inflammation and dysregulated angiogenesis.

## Materials and Methods

For additional materials and methods information (microPET, instrumentation, orthotopic pancreas cancer study, HRG fluorescent labeling, NanoPro isoelectric focusing), see Methods S1.

### HRG expression vector, transfection and protein purification

Full-length human and murine HRG cDNA (hHRG; ENST00000232003 and mHRG; ENSMUST00000023590), including the signal sequence were cloned into the pCEP-Pu2 expression vector and used for transfection of human embryonic kidney HEK293-EBNA cells. To avoid contamination with bovine serum-derived HRG, serum-replacement medium, TCM (ICN Biomedicals) was used. HRG was purified using Ni-NTA agarose (Qiagen). Protein-containing fractions were pooled and dialyzed against PBS (pH 7.4). The protein preparation lacked lipopolysaccharide (endotoxin) contamination as determined using a sensitive chromogenic endotoxin quantification kit (Pierce).

### Amino acid analysis

The procedure used for the quantitative analysis of amino acid composition was based on the classical system of Spackman, Moore and Stein [19], whereby the amino acids are separated by cation-exchange chromatography on sulfonated polystyrene resins and detected in the effluent by means of a ninhydrin reaction. Importantly, the yields for the amino acid residues histidine and proline were close to 100%.

### ELISA

The murine HRG ELISA was performed using a rat anti-mouse (m)HRG antibody (capture, R&D systems, MAB1905, 1 µg/ml), biotinylated goat anti-mHRG (detection, R&D systems, BAF1905, 9.6 ng/ml), Streptavidin-HRP (Vector Labs, SA-5004, 2 µg/ml) and recombinant standard produced in-house. The human HRG ELISA was performed using a rabbit anti-human (h) HRG (capture, produced in-house, HRG-0119, 1∶1000), polyclonal mouse anti-hHRG (detection, AbNova, H00003273-B01P, 20 ng/ml, 1∶500), biotinylated anti-mouse IgG antibody (Vector Laboratories, SA-5004, 2 µg/ml) and recombinant standard produced in-house.

### Labeling chemistry

Forty micrograms of HRG, 0.5 µg/µl in PBS, was mixed with ^125^I-iodide (15–80 MBq; Perkin Elmer) in an IodoGen coated tube (Pierce), incubated for 30 min and purified on NAP-5 columns (GE Healthcare). The yield and radiochemical purity was analyzed using 150–771 DARK GREEN, Tec-Control Chromatography strips (Biodex Medical Systems) and eluted using 80% acetone. Bovine (Fraction V, fatty acid free, Roche) was similarly radioiodinated. Before labeling with ^124^I for microPET imaging, ^124^I-sodium iodide (19 MBq) was mixed with 0.04 nmol of non-radioactive sodium iodide and incubated for 20 min. Further labeling was performed as described above.

### Heparin-binding assay

Maxisorp 96-well plates (Nunc-Immuno plate, Nunc) were coated with 50 mg/ml heparin sodium salt (Sigma) in Tris-buffered saline overnight at 4°C, and washed 3 times with PBS/0.05% Tween 20 (TPBS) before use. Nonspecific binding was blocked with 10 mg/ml pluronic solution (Pluronic F108NF prill poloxamer 338, 30085231, BASF) in PBS for 1 h at 37°C. HRG (100 or 500 ng/ml) in PBS, 0.5% BSA was added to the wells for 1.5 h at room temperature (RT). Radiolabeled HRG was added at a concentration of 10 nM in PBS containing 10 µM ZnCl_2_ to the six wells, coated with heparin. Blocking with non-labeled HRG (66 nM) in triplicate wells was initiated 15 min before addition of ^125^I-HRG. Samples were incubated for 1 h at RT, followed by washing 3 times in TPBS. The radioactivity in each well was then measured.

### Chemotaxis assay

Chemotaxis of human umbilical vein endothelial cells (HUVECs, American Type Tissue Collection) was assessed using a modified Boyden chamber, as described earlier [17], with 8-µm micropore polycarbonate filter (Nuclepore track-etch membrane, 155846, Whatman) coated with type 1 collagen solution at 100 ng/ml (PureColTM, 5410, Biomaterials). Cells starved overnight in 1% FCS were trypsinized and resuspended at 4.0×10^5^/ml in MV-2 medium (Promocell GmbH), 0.25% BSA, and trasylol (Aprotinin, 495184, Bayer Healthcare) at 1,000 KIE/mL. The cell suspension was added to the upper chamber and the chemoattractant, human vascular endothelial growth factor A165 (VEGF; Peprotech), was added at 10 ng/ml to the lower chamber. HRG (100 ng/ml), iodinated with non-radioactive iodide (Merck) as described above, was added to both chambers. As a control, HRG was omitted to one set of cells. Another set of cells was incubated with HRG not exposed to the labeling procedure. After 5 h at 37°C, cells that had migrated through the filter were stained with Giemsa and counted using the Image J software (http://rsbweb.nih.gov/ij/). All samples were tested in at least six wells for each condition.

### Animal experimentation

Studies on C57BL/6 wild type mice (8–10 weeks; 18.6±1.9 g; Taconic M&B) were carried out in strict accordance with the ethical permit provided by the Committee on the Ethics of Animal Experiments of the University of Uppsala (Permit Numbers: C59/10, and C 224/10). Mice were anaesthesized by isoflurane inhalation (Forene, Abbott Laboratories, Abbott, IL). All efforts were made to minimize suffering and mice showing signs of pain or discomfort were removed, supervised by the University veterinarian.

For tumor challenge, mice were inoculated with 10^6^ T241 fibrosarcoma cells subcutaneously into the left flank. Tumors were measured with a caliper once every 2 days, in a blind procedure, and volumes were calculated by the formula: Tumor volume  = 0.52× (D×d2), where d is the minor tumor axis and D is the major tumor axis. On day 11 (early stage) or day 21 (late stage) after injection, mice were sacrificed and plasma, liver and tumors were harvested.

To assess HRG degradation in blood, we intravenously injected 2.5 µg of ^125^I-labeled HRG in in healthy and 4T1 tumor bearing mice (1 cm^3^ tumors, approximately 20 days after inoculation of tumor cells). After 2 h, ^125^I-radioactivity in the plasma fraction was precipitated by adding ice-cold trichloroacetic acid (TCA) to a final concentration of 10%, to precipitate fragments larger than approximately 5 kDa. Counts per minute (cpm) were measured on the precipitated fraction.

### Comparative biodistribution of ^125^I-hHRG and ^125^I-albumin in C57BL/6 naive and tumor-bearing mice

Six groups of C57BL/6 mice were injected intravenously (i.v.) with ^125^I-hHRG (30 kBq in 100 µl PBS per mouse). Four C57BL/6 mice were used for each data point. Each protein injection dose was adjusted to 5 µg/mouse by adding non-labeled hHRG. Bovine ^125^I-albumin, a size-matched comparator protein, was injected into four groups (30 kBq/5 µg in 100 µl PBS per mouse). Mice were euthanized by an intraperitoneal (i.p.) injection of ketamine/xylazine. Blood and organ samples were collected, weighed and their radioactivity content determined. The organ uptake values were calculated as percent injected dose per gram tissue (% ID/g). Aliquots of blood samples were separated into low- and high-molecular-weight fractions (<and>5 kDa) using size-exclusion NAP-5 columns, pre-equilibrated with 2% BSA in PBS. The percentage of radioactivity in the high-molecular weight fractions was calculated. When indicated, C57BL/6 mice were inoculated with T241 fibrosarcoma cells subcutaneously (sc) into the left flank (1×10^6^ cells/animal). Biodistribution analyses were performed using mice with 0.21±0.06g tumors (day 11 after injection). Five groups of mice were injected iv with ^125^I-mHRG (30 kBq/5 µg in 100 µl PBS per mouse). Statistical significance was calculated using unpaired t test, p<0.05 was considered significant.

### Tissue microarrays

Tissue microarrays (TMAs) of healthy and malignant tissues, containing multiple samples from healthy tissue, different stages of human colorectal carcinoma, adenoma, and distant metastasis, produced by the Human Proteome Atlas (HPA) facility (www.proteinatlas.org), were stained using anti-HRG antiserum (0119) or an antibody against CD45 [17], [20]. TMA sections were scanned by high resolution scanners (ScanScope XT, Aperio Technologies), separated into individual spot images, and evaluated by experienced pathologists. Ethical permit (Ups 02-577; no 2011/473) to use anonymized, de-coded (i.e. non-traceable) human paraformaldehyde-fixed normal or tumor tissue for generation of tissue slides or TMAs was granted by the Uppsala ethical review board in full agreement with the Swedish Ethical Review Act.

### Binding of ^125^I-HRG to U937 cells

Vitamin D3-treated monocytic U937 cells [21] (a kind gift from Dr. Fredrik Öberg, Uppsala University) were counted, resuspended in PBS and 10^6^ cells were distributed per tube in Eppendorf tubes precoated o/n at 37°C with Pluronic solution to minimize unspecific binding. Cells were treated for 1 h at 37°C with heparitinase (2 UI/ml final concentration; Sigma), where indicated. Competition with unlabeled HRG was performed using a 10 fold molar excess; 100 nM (7.5 µg/ml) for 30 min at 4°C. Radiolabeled ^125^I-hHRG (10 nM final concentration (0.75 µg/ml) was added to each sample and incubated for 1 h at 4°C. Cells were washed, resuspended in 200 µl of PBS and the radioactivity was measured using an automated γ-counter. All experiments were performed in triplicates.

### Measurement of HRG transcript levels by qPCR

Snap-frozen livers from mice with and without T241 fibrosarcoma were lysed, followed by RNA isolation using Quiagen RNA isolation kit. Complementary DNA (cDNA) was transcribed using Superscript II Reverse Transcriptase kit (Invitrogen). Gene expression was detected by using the following Taqman Probes (Applied Biosystems): Mm00504391_m1 for HRG and Mm01324427_m1 for HPRT.

### CSF1-neutralizing antibody treatment

8–10 weeks old male C57Bl/6 mice were treated by i.p. injection with 1 mg per mouse of neutralizing anti-CSF1 antibody (Clone 5A1, BioXCell, USA) or isotype control (BE0088, BioXCell, USA) and 4 days later with 0.5 mg/mouse antibody given i.p. as described by DeNardo et al [22]. At day 7 after initiation of treatment animals were administered radiolabeled HRG for biodistribution analysis. Blood was taken from control animals prior to treatment, at 3 and 7 days after treatment, for endogenous HRG analysis. After 7 days, organs were harvested for immunofluorescent analysis using anti-CD115 (sc-692, Santa Cruz Biotechnology, USA), anti-CD68 (MCA1957AbD Serotec, USA), anti-Ly6G (551459, BD Biosciences, USA), and appropriate fluorescently labeled secondary antibodies (Life Technologies). Immunoblotting to show plasma protein levels was done using anti-Fibrinogen (GAM/Fbg/7S, Nordic Immunology, The Netherlands) and anti-VWF (A0082, Dako, USA) antibodies.

### Micro-autoradiography of tissues

Tumor bearing and naive C57BL/6 mice were injected with ^125^I-mHRG (5 µg, 5 MBq, in 100 µl PBS). Mice were euthanized 15 min post-injection and spleens and tumors were dissected and snap-frozen. Tissues from non-injected mice were used as controls. Frozen sections (10 µm) were cut with a cryostat microtome. Two consecutive sections of each organ were mounted on Menzel Super Frost plus glass slides, fixed in methanol for 10 min, equilibrated in PBS, incubated with 3% hydrogen peroxide for 10 min, blocked in 3% BSA/0.1% Triton X-100/5% FCS in PBS (blocking solution) for 1 h and incubated with primary antibodies diluted in blocking solution overnight at 4°C. The following primary antibodies were used: rat anti-mouse CD31 (BD Pharmingen) and goat anti-mouse CD45 (R&D). Immunolabeled cells were detected with the Tyramide Signal Amplification (TSA) system (PerkinElmer) and the AEC detection kit (Vector). Samples were covered with photographic emulsion (NTB2, Eastman Kodak Company) and exposed for about 8–12 weeks before development. Slides were visualized using a Nikon Eclipse E100 microscope.

### Statistical analyses

Statistical analyses were performed using Student's t-test (GraphPad Prism 6.0); p<0.05 was considered significant.

## Results

### Radiolabeled HRG retains biological activity and is stable in mouse plasma

**Figure 1:**
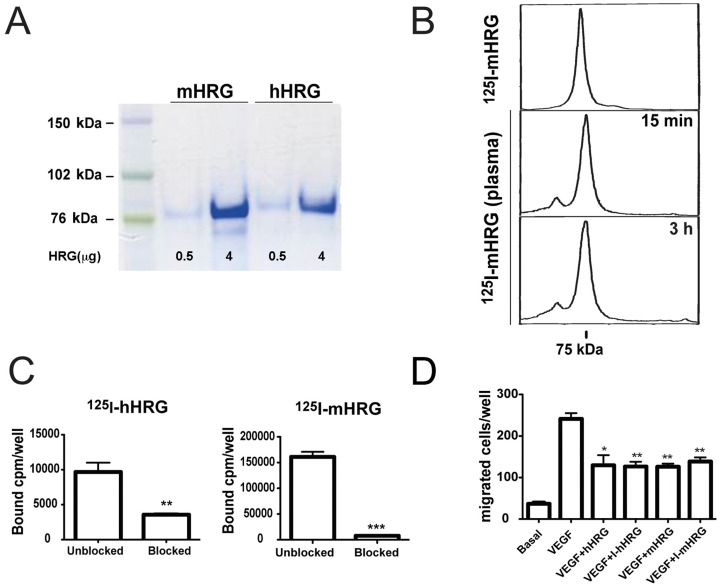
Purification and bioactivity of radiolabeled HRG. A. Coomassie brilliant blue-stained SDS-PAGE showing purified mHRG and hHRG protein (0.5 µg and 4 µg/lane). B. SDS-PAGE analysis of freshly radiolabelled ^125^I-mHRG (top) and after incubation in murine plasma at 37°C for 15 min (middle) and 3 h (bottom) visualized using Cyclone Storage Phosphor system. C. Binding of ^125^I-hHRG and ^125^I-mHRG to heparin-coated plastic *in vitro* in the presence (blocked) and absence (unblocked) of unlabeled HRG; *p<0.05; Student's t test. D. Inhibition of human umbilical vein endothelial cell migration towards VEGFA by non-radioactive iodinated hHRG and mHRG (HRG-I). *p<0.05; **p<0.01; Student's t test.

In order to study the biodistribution and cellular uptake of HRG, we purified recombinant hHRG and mHRG from overexpressing HEK293-EBNA cells (Figure 1A) to endotoxin-free preparations of greater than 97% purity, as estimated from amino acid analysis (data not shown). hHRG and mHRG preparations were labeled with ^125^I using a mild IodoGen procedure resulting in less than one iodine atom per HRG molecule and a yield exceeding 90%. Size-exclusion purification provided radiochemical purity over 99%. The integrity of radiolabeled protein was confirmed by denaturing SDS-PAGE showing a single radioactivity peak (Figure 1B top panel) with a migration rate matching that of unlabeled HRG. To ensure that the labeling did not interfere with the biological activity of HRG, we determined its capacity to bind heparin (Figure 1C) and to inhibit chemotaxis of endothelial cells towards VEGF (Figure 1D) before and after cold iodination. Importantly, both assays demonstrated preserved bioactivity of radiolabeled hHRG and mHRG.

Incubation of ^125^I-mHRG in mouse plasma at 37°C for 15 min or 3 h (Figure 1B) resulted in less than 1% release of low molecular weight radioactivity. The majority of the radiolabeled HRG appeared in one narrow peak; less than 5% of the radioactivity was associated with a high molecular weight fraction (>75 kDa). We conclude that radiolabeled HRG was a bioactive, homogenous preparation that remained stable for at least 3 h in plasma, *in vitro*.

### Biodistribution of radiolabeled HRG shows rapid uptake and blood clearance

**Figure 2:**
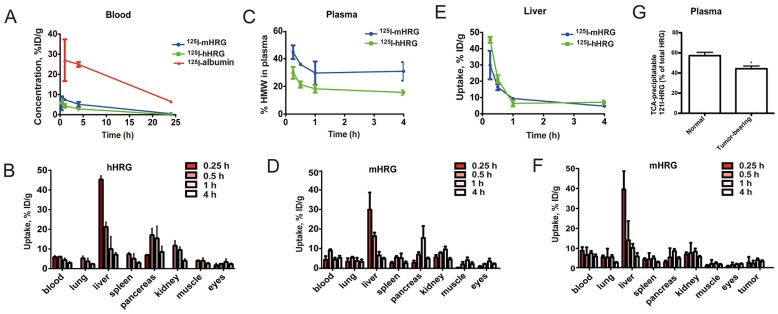
Unusually rapid biodistribution of radiolabeled HRG. A. Blood kinetics of ^125^I-albumin, ^125^I-hHRG and ^125^I-mHRG in C57BL/6 mice. (n = 4/time point). B. Biodistribution of ^125^I-hHRG in selected C57BL/6 mouse organs. C. Percentage of radioactivity in blood plasma, associated with a high molecular-weight fraction (>5 kDa). D. Biodistribution of ^125^I-mHRG in selected organs of naive C57BL/6 mice. E. Liver uptake of ^125^I-mHRG and ^125^I-mHRG. F. Biodistribution of ^125^I-mHRG in selected organs of T241 fibrosarcoma-bearing C57BL/6 mice. G. TCA-precipitable ^125^I-radioactivity in plasma after 2 h of circulation in naive and tumor-bearing mice injected with ^125^I-mHRG.

To study the biodistribution of HRG, ^125^I-hHRG was injected into the tail vein of C57BL/6 mice and the distribution of radioactivity in blood and a range of organs, was followed over time (Figure 2A, B, Table 1). For comparison, ^125^I-albumin was injected in a parallel cohort of mice. The biodistribution of ^125^I-albumin was typical for a protein of its size (67 kDa) [23], i.e. larger than the cut-off for glomerular filtration, with slow blood clearance and distribution among organs. In contrast, the disappearance of radiolabeled hHRG from blood and distribution to organs was very quick. Already at 1 h post-injection, the concentration of ^125^I-hHRG in blood was six-fold lower than that for ^125^I-albumin (Figure 2A). Accordingly, the level of ^125^I-hHRG exceeded that of ^125^I-albumin in pancreas (8.4-fold), stomach (4.3-fold), muscle (4.1-fold) and liver (2.5-fold) at this time point (Table 1).

**Table 1:** Comparative biodistribution of ^125^I-hHRG and ^125^I-albumin in female C57Bl/6 mice.

	0. 5 h	1 h		4 h		8 h		24 h	
Organ	hHRG	hHRG	Albumin	hHRG	Albumin	hHRG	Albumin	hHRG	Albumin
Blood	6.1±0.2	4.4±0.9*	27±10	2.9±0.6*	25±1	0.6±0.1*	16±2	0.09±0.01*	6.5±0.6
Heart	3.6±0.5	2.6±0.8	6±3	2.0±0.5*	6.3±0.8	0.5±0.1*	4.2±0.6	0.06±0.02*	1.7±0.3
Lung	5±1	4±2	8±3*	2.3±0.3	8.2±0.4	0.5±0.1*	5.9±1.0	0.079±0.004*	2.8±0.4
Saliv gland	7±1	6±3	5±3	2.1±0.4*	7±2	0.8±0.7*	3.5±0.5	0.07±0.01*	3.0±0.5
Liver	21±2	10±6	4±1	7±1*	4.2±0.3	3±1	2.9±0.7	1.9±0.2*	1.2±0.3
Spleen	7.4±0.8	5±2	4±2	3.0±0.5*	4.1±0.7	0.9±0.2*	2.1±0.7	0.38±0.09*	1.2±0.2
Pancreas	17±3	15±6*	1.8±0.6	9±3*	2.7±0.6	2±1	2.1±0.5	0.17±0.0410*	1.2±0.4
Stomach	12±2	12±7	2.8±0.6	5±3	9±1	0.4±0.4*	2.7±0.3	0.13±0.01*	1.3±0.3
S intestine	6±1	3.7±0.9	2.4±0.6	4±1	3.4±0.3	0.7±0.4*	2.3±0.4	0.08±0.02*	0.9±0.3
L intestine	4.1±0.5	3±1*	1.5±0.3	2.0±0.4*	2.7±0.2	0.6±0.3*	2.0±0.3	0.08±0.0210*	1.0±0.2
Kidney	12±3	10±2*	5±2	4.1±0.9*	6.4±0.2	1.7±1.0*	3.8±0.6	0.36±0.09*	1.7±0.4
Adrenals	4.8±0.7	4±1	6±2	2.1±0.3*	5.7±0.9	0.6±0.3	3±1	0.4±0.2*	2±1
Uterus	5.9±0.9	5±2	3±3	2.8±0.8*	6±2	0.74±0.04*	4±1	0.06±0.01*	2.1±0.7
Skin	4.1±0.5	3.2±1.0	3.5±1.0	2.3±0.4*	4.5±0.6	0.6±0.2*	4.1±0.6	0.2±0.1*	1.9±0.2
Muscle	4.0±0.3	4±1*	1.0±0.3	2.6±0.5*	1.6±0.3	0.7±0.1*	1.6±0.4	0.06±0.01*	0.6±0.1
Bone	4.3±0.4	3±1	2.2±0.9	2.4±0.4	2.5±0.1	0.7±0.1*	1.7±0.5	0.16±0.08*	0.9±0.1
Eyes	2.5±0.2	2.6±1.0*	1.1±0.5	2.4±1.0*	1.1±0.3	1.2±0.4	0.9±0.3	0.33±0.04	0.7±0.3
Brain	2.0±0.4	2.0±0.6*	0.50±0.08	1.2±0.4*	0.5±0.1	0.4±0.1	0.29±0.06	0.027±0.0030	0.16±0.02
Intestines§	4.3±0.5	5±3	1.7±0.7	3±1	2.8±0.5	0.8±0.1*	2.1±0.7	0.30±0.04*	0.9±0.1
Carcass§	43.4±3.0	55±11*	37±7	24±5*	38±4	8±2*	27±3	1.2±0.1*	13±2

Uptake is expressed as % ID/g and presented as an average value from 4 animals ± standard deviation.

§) Data for intestines with content and carcass are presented as %ID per whole sample.

* Significant (*p*<0.05) difference between hHRG and albumin at this time point.

The pattern of rapid uptake and distribution of radiolabeled HRG was established already at 15 min after injection. At this time, the level of ^125^I-hHRG in the blood was 5.9±0.7% of the injected dose/gram tissue (ID/g), and the liver uptake was 45.3±0.7% ID/g (Figure 2B). There was a subsequent accumulation of radioactivity in spleen, pancreas and kidney. At 1 h after injection, only 4.4±0.9% ID/g of HRG remained in the plasma (Table 1) and, of this relatively small pool, more than 80% was fragmented to low-molecular weight peptides (less than 5 kDa) based upon size exclusion chromatography (Figure 2C). This suggested very rapid, first-pass, binding and internalization of ^125^I-hHRG in the liver with subsequent degradation and release of radiocatabolites back to the circulation. MicroPET imaging revealed a similar pattern with very rapid liver uptake of radiolabeled HRG followed by the appearance of radioactivity in the spleen, pancreas and kidney (Figure S1).

Although the biodistribution of ^125^I-mHRG (Figure 2D, Table 2) showed differences in uptake as compared to ^125^I-hHRG, the general pattern for the two species agreed on the very rapid blood clearance (Figure 2A, C–E). Thus, about 70% of the total radioactivity in plasma was of 5 kDa or less at 1 h after injection of ^125^I-mHRG (Figure 2C), as judged from size-exclusion chromatography. Interestingly, the radioactivity uptake in eyes exceeded that of the brain by at least 3-fold, although both organs were protected from the general circulation through the blood-brain and the blood-retina barriers, respectively (Table 2).

**Table 2:** Biodistribution of ^125^I-mHRG in normal and tumor-bearing C57Bl/6 mice.

	0.25 h		0.5 h		1 h		4 h		24 h	
Organ	Naïve	Tumor	Naïve	Tumor	Naïve	Tumor	Naïve	Tumor	Naïve	Tumor
Blood	4±2*	9±2	8.9±0.7#	7±4	7.5±0.4#	8±1	5±1#	6±1	0.5±0.1#	0.5±0.1
Lung	3±2	5.6±0.9	5.3±0.5	6±4	5.0±0.6	6±1	3±1	2.9±0.3	0.3±0.2	0.4±0.2
Liver	30±9	40±9	16±2#	14±10	9.4±0.5	10±2	4.7±0.6#	6±2	1.0±0.5#	1.3±0.6
Spleen	2.5±0.8*	4.4±0.7	5.4±0.8#	5±3	4.8±0.6	5±1	2.5±0.9	3.0±0.4	0.6±0.2	0.3±0.2
Pancreas	2.7±1.0	3.0±0.8	7±1#	6±5	7±2	9±1	4.9±0.6#	5.0±0.7	0.20±0.06	0.3±0.3
Kidney	6±1	7±1	7.8±0.4#	8±5	6.7±0.2#	8±2	4.4±0.8*	5.7±0.5	0.49±0.10	0.7±0.1
Skin	2.2±1.0	2.7±0.8	4.7±0.1	4±2	4.6±0.5	5±2	2.8±0.6	2.7±1.0	0.5±0.2	0.23±0.01
Muscle	0.19±0.06*	1.3±0.4	2±1#	2.2±2.1	2.52±0.06	2.5±0.6	2.1±0.9	1.8±0.5	0.11±0.01*	0.09±0.01
Eyes	0.7±0.4	1.0±0.3	2.1±0.1#	2±1	2.3±0.2	2.0±0.3	2.1±0.3	2.2±0.4	0.16±0.02#	0.3±0.1
Brain	0.2±0.1	0.3±0.1	0.76±0.09#	0.6±0.3	0.93±0.08#	1.0±0.1	0.59±0.03#	0.7±0.1	0.029±0.006	0.03±0.01
Tumor		3±3		2±1		4.2±0.8‡		3.5±0.6‡		0.21±0.05‡
GI tract§	12±11	7±2	15±2#	16±10	14.5±0.4#	14±9	12±1#	10±3	0.4±0.1	0.59±0.10
Carcass§	27±17	28±7	37.0±0.6‡	46±7	38±1	32±16	25±3	27±5	2.4±0.4#	2.0±0.3

Uptake is expressed as % ID/g and presented as an average value from 4 animals ± S.D.

“Naive” and “Tumor”, indicate healthy mice and mice challenged with subcutaneous T241 fibrosarcomes, respectively.

§ ) Data for intestines with content and carcass are presented as %ID per whole sample.

*) Significant (*p*<0.05) difference between normal and tumor bearing mice at this time point.

# ) Significant (*p*<0.05) difference between hHRG and mHRG at this time point.

‡ ) Significant (*p*<0.05) difference between tumor and muscle at this time point.

The biodistribution of ^125^I-mHRG was also studied in T241 fibrosarcoma-bearing mice 11 days after tumor inoculation (Figure 2F) when tumors were clearly established. Overall, there were small or no differences in the biodistribution between naive control mice and tumor-bearing mice (Figure 2D, F; Table 2). Also, during the duration of the study, there was no significant accumulation of radioactivity in the tumor over time. However, there were indications of increased degradation of HRG in tumor-bearing mice as the extent of TCA-extracted ^125^I-mHRG in plasma was reduced when a tumor was present (Figure 2G).

We conclude that purified, bioactive, radiolabeled HRG was rapidly cleared from the blood and distributed in organs with a similar pattern in naive and tumor-bearing mice, and that the majority of this exogenous pool was degraded to very small fragments. The extent of this degradation was enhanced in tumor-bearing mice. These data indicate that endogenous HRG is rapidly turned over in normal and tumor-bearing mice.

### HRG steady state levels remain constant during tumor disease in mice and humans

**Figure 3:**
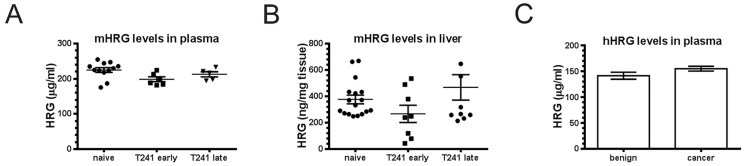
Stable HRG levels in livers and plasma from T241 fibrosarcoma-bearing mice and plasma in CRC patients. ELISA showing HRG levels in plasma (A) and livers (B) from naïve and T241 fibrosarcoma-bearing mice terminated at early (day 11 after inoculation) or late (day 21) stages. C. ELISA showing HRG levels in the plasma of healthy individuals (n = 66) and patients with CRC stage 1–4 (n = 146). p = 0.103, t-test.

To determine whether the increased turnover of HRG observed in tumor-bearing mice bore any significance for steady-state levels of HRG, we employed ELISAs specific to either the mouse or human HRG protein. Analyses of HRG levels in the plasma and liver of T241 fibrosarcoma bearing mice was assessed at 11 or 21 days post-implantation. As shown in Figure 3A, the levels of HRG in plasma remained remarkably stable irrespective of tumor disease. The same consistent HRG levels were seen in mice with orthotopic Panc02 pancreatic cancer (Figure S2). Analysis of liver lysates showed a trend towards decreased HRG levels during early stage disease (Figure 3B), which subsequently returned to the levels seen in unchallenged mice. The liver HRG mRNA levels on the other hand, increased slightly during early stage disease, possibly in a compensatory manner (Figure S3).

To confirm that the data obtained using mouse tumor models reflects the situation in human cancer, we measured HRG levels in plasma from colorectal cancer (CRC) patients at different stages of progression compared to age- and sex-matched healthy control patients. As seen in Figure 3C, there was no significant difference in HRG plasma levels between healthy controls compared to CRC patients. Comparison of the different CRC stages individually to the controls did also not show any disease-related variation (data not shown).

### HRG immunoreactivity in human cancer

**Figure 4:**
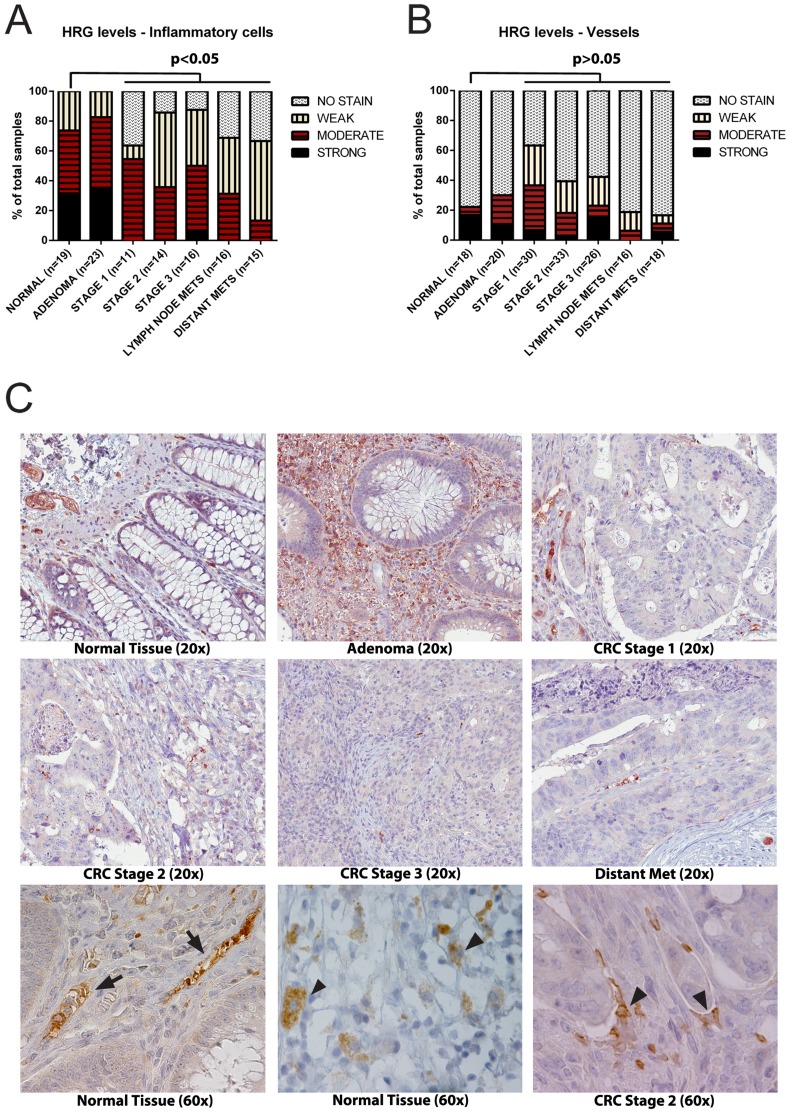
HRG detected by IHC of CRC tumor tissue arrays. A. Scoring of HRG IHC signals associated with inflammatory cells in CRC arrays from strong to no signal. Statistical analysis; p<0.05 was considered significant. The number, n, of biopsies were; normal = 10, adenoma = 10, stage 1 = 20, stage 2 = 20, stage 3 = 20, lymph node metastasis = 10, distant metastasis = 10. B. Scoring of HRG IHC signals associated with vessels, as above. C. Upper and middle row of panels: Representative images of the HRG IHC signals from the indicated categories at 20× magnification. Lower row of panels: Representative images of the HRC IHC signals in CRC at 60× magnification. Arrows indicate typical vessel-associated HRG signals in normal colorectal tissue (left) and in inflammatory cells in normal tissue (middle) and in stage 2 CRC (right).

Previously, we reported that HRG immunodetection is decreased in a range of human cancers [1]. Our observation of increased HRG degradation, related to the presence of a tumor (Figure 2G), may explain this finding. Consequently, we sought to explore in more detail, the deposition of HRG in human clinical CRC samples. To this end, we used the anti-HRG antibody (0119) to probe CRC biopsy arrays by immunohistochemical staining. Immunoreactivity was scored from “no stain” to “strong” using standard pathology procedures, in biopsies from normal tissues, adenomas, different CRC stages, lymph node metastases and distant metastases (Figure 4). HRG immunoreactivity occurred in the stroma outside vessels, mainly on inflammatory cells (Figure 4A) and in the vasculature and perivascular area (Figure 4B; C). Blinded scoring demonstrated strong or moderate stromal immunostaining for HRG in the majority of normal (healthy) and adenoma tissues. In contrast, there was a dramatic decrease in HRG immunostaining intensity in the stroma of tumor tissue samples from CRC as well as lymphatic and distant metastases (Figure 4A, C). In 90% of the distant metastasis, HRG immunoreactivity in the stroma was weak or not detected.

We hypothesized that the decrease in HRG immunoreactivity from the stroma associated with cancer progression, may be correlated with an altered inflammatory status. We therefore performed immunohistochemical staining for CD45, a pan-inflammatory cell marker, on the CRC tumor tissue arrays. As shown in Figure S4, scoring for the level of CD45 immunoreactivity decreased with progression of the disease and was minimal in the distant metastases. The decreased density in inflammatory cell infiltrate with increased CRC stage is in agreement with previous reports [24].

Combined, these data indicated 1) that the levels of HRG in the vasculature and the perivascular area is unaffected by tumor progression reflecting persistent steady state levels of HRG and 2) that HRG may be associated with inflammatory cells and that these cells may contribute to how HRG is distributed in tissues. There was, however, no significant correlation in the decline in immunoreactivity patterns between sample-matched HRG and CD45 immunostaining (data not shown), possibly indicating that HRG was associated with a subset of inflammatory cells, alternatively, with cells at a particular stage of activation.

### *In vivo* tracing of ^125^I-mHRG shows accumulation on inflammatory cells and blood vessels

**Figure 5:**
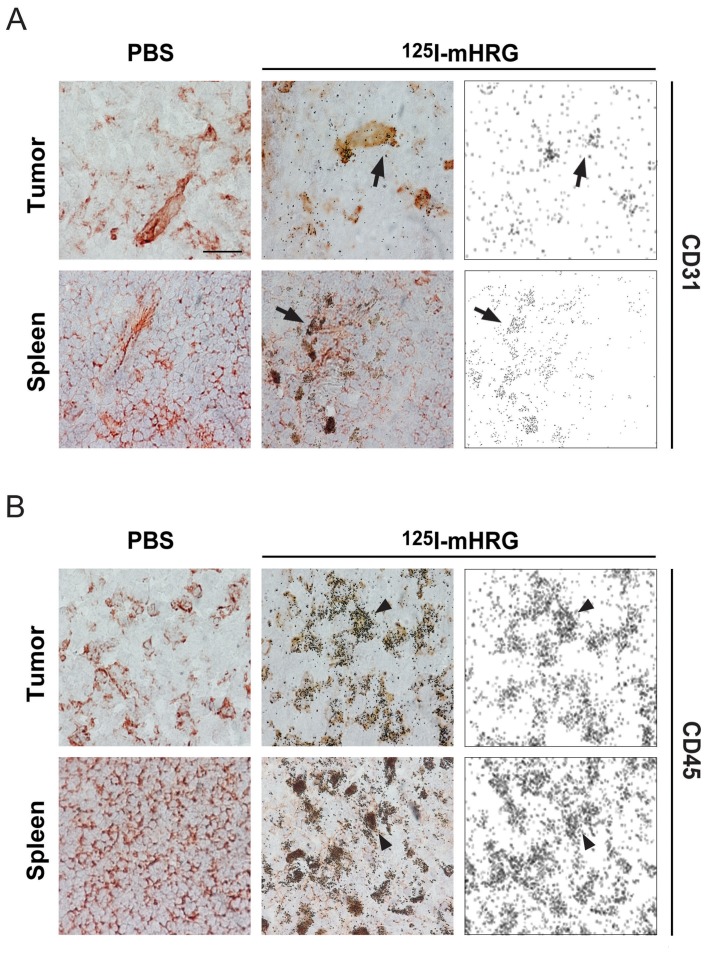
Radiolabeled HRG homes to the perivascular area and to inflammatory cells. Micro-autoradiography of ^125^I-mHRG in the spleen and T241 fibrosarcomas (Tumor) tissue at 15 min post injection of radiolabeled HRG. A. Panels show tumor and spleen tissues from mice injected with PBS (left) or ^125^I-mHRG (middle and right). Immunohistochemical staining with CD31 antibodies show endothelial cells colocalized with ^125^I-mHRG in the middle panels (arrows). Panels to the right show the retention of ^125^I-mHRG alone. B. Panels as above but immunohistochemical staining with CD45 antibodies to identify leukocytes. Arrowheads in the middle panel indicate colocalization of CD45-positive leukocytes and ^125^I-mHRG. Panels to the right show the retention of ^125^I-mHRG alone.

To further identify the cell type(s) that bind HRG *in vivo*, we injected bioactive ^125^I-mHRG or an equal volume of PBS, into T241-bearing C57BL/6 mice. After 15 min circulation, spleen and tumor tissues were harvested and processed for micro-autoradiography (Figure 5). In T241 tumors and spleens, ^125^I-mHRG accumulation occurred on specific cell types, which were identified as endothelial and inflammatory cells, using immunohistochemical staining of the autoradiographed sections for CD31 and CD45, respectively. Figure 5A shows that ^125^I-mHRG colocalized with CD31-positive blood vessels. The accumulation of ^125^I-mHRG on CD45-positive cells (Figure 5B) was extensive.

Parallel macro-autoradiography in which ^125^I-mHRG was incubated directly on spleen and tumor sections in the absence and presence of a 100-fold molar excess of unlabeled HRG, showed that binding of ^125^I-mHRG was efficiently blocked by unlabeled HRG (data not shown).

### ^125^I-hHRG binds directly to U937 monoblastoid cells

**Figure 6:**
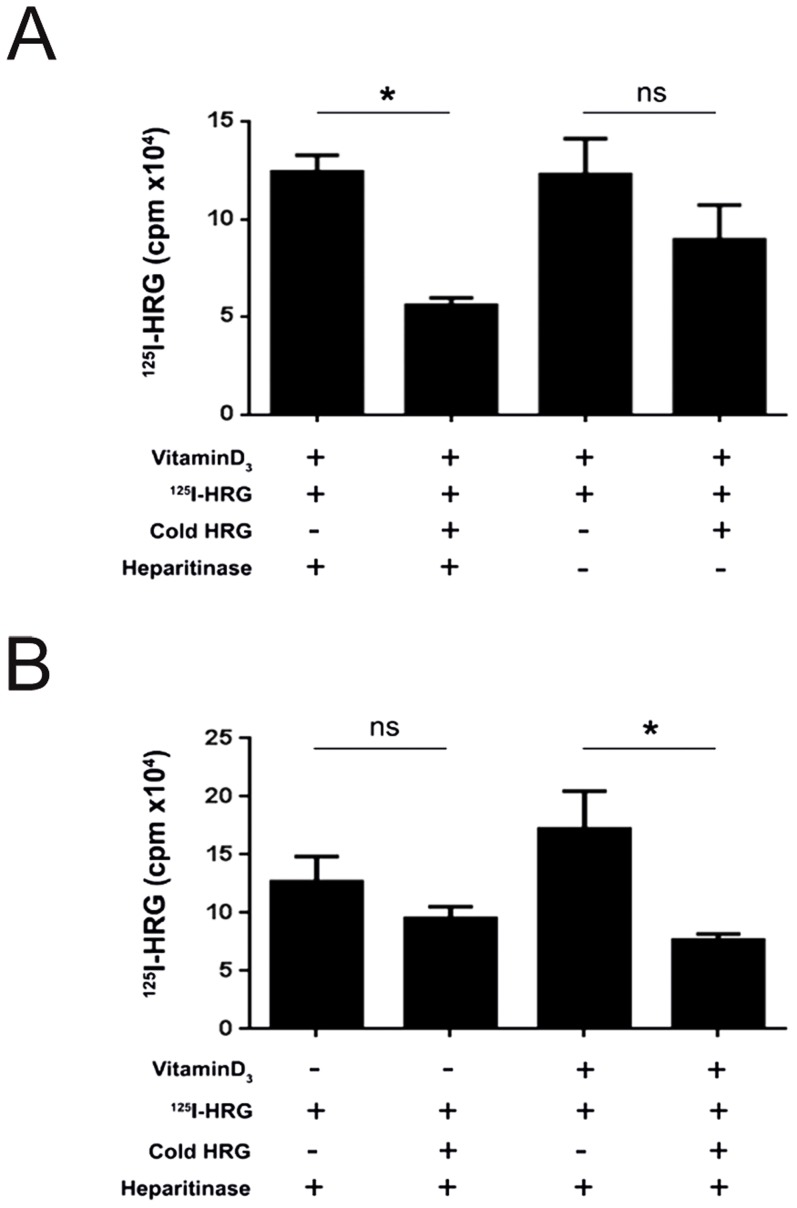
Binding of radiolabeled HRG to U937 cells. A. Binding of ^125^I-mHRG to differentiated U937 cells (treated with 1a, 25(OH)_2_D_3_ (VitD3) for 24 h) was competed with a 10-fold concentration of unlabeled (cold) HRG on cells treated or not with heparitinase. *; p<0.05, Student's t-test. ns; not significant. B. Binding of ^125^I-mHRG to undifferentiated or differentiated U937 cells (treated with 1a, 25(OH)_2_D_3_ (VitD3) for 24 h) was competed with a 10-fold concentration of unlabeled (cold) HRG. All cells were treated with heparitinase. *; p<0.05, Student's t-test. ns; not significant.

To explore the capacity of inflammatory cells to bind HRG, we selected the CD45-positive human U937 cell line, which differentiates along the monocyte/macrophage lineage in response to vitamin D3 (vitD3) [21], [25]. Binding of ^125^I-hHRG to U937 cells, treated with vitamin D3 for 24 h, was saturable and specific as it was competed by a 10-fold excess of unlabeled (“cold”) HRG on cells that had been treated with heparitinase to remove cell surface heparan sulfate (Figure 6A). In cells not treated with heparitinase, binding of ^125^I-hHRG was not significantly competed by cold HRG. These data indicate that U937 cells express a cell surface protein that binds HRG in a saturable, non-heparan sulfate-dependent manner. Moreover, specific binding of ^125^I-hHRG occurred to vitD3-differentiated U937 cells, but not to undifferentiated cells (Figure 6B). We further used the RAW 264.7 mouse leukaemic macrophage cell line to show binding and uptake of fluorescently labeled HRG (Figure S5). We conclude that HRG binds specifically to cells of the monocyte/macrophage lineage.

### Suppressed monocyte differentiation leads to decreased HRG uptake and to accumulation of HRG in the blood

**Figure 7:**
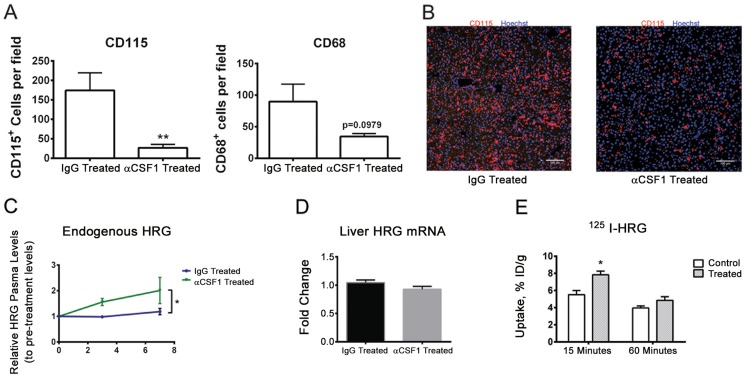
Decreased HRG turnover with anti-CSF1 antibody treatment. A. Number of CD115^+^ and CD68^+^ cells in livers of mice at day 7 of treatment of C57BL/6 mice with anti-CSF1 neutralizing antibody as compared to treatment with isotype-matched control. n = 4 for each treatment. **p<0.01, or as indicated. Student's t-test. B. Morphology of CD115^+^ cells (red) in the liver with anti-CSF1 antibody treatment or with isotype-matched control IgG antibody treatment for 7 days. Blue indicates nuclei stained with Hoechst 33342. Bar; 100 µm. C. Levels of endogenous HRG in blood in mice treated with the anti-CSF1 antibody or an isotype-matched control at different time points. N = 4 for each treatment. **p<0.01, Student's t-test. D. Liver *hrg* transcript levels at day 7 of anti-CSF1 or control IgG treatment. E. Blood kinetics of ^125^I-mHRG after tail vein injection of C57BL/6 mice and circulation for 15 min or 60 min, performed at day 7 of treatment with anti-CSF1 antibody or isotype-matched control (n = 4/time point). *p<0.05, Student's t-test.

In order to test the role of mononuclear phagocytes in the uptake and turnover of HRG, we treated C57BL/6 mice with a neutralizing anti-colony stimulating factor (CSF)-1 antibody (Ab). CSF1 regulates the survival, proliferation and maturation of mononuclear phagocytes, preferentially monocytes and macrophages; neutralization of CSF1 is known to result in reduced numbers of CD115 (CSF1R)-positive cells [26], [27], [28]. As shown in Figure 7A, the number of CD115^+^ cells in the liver decreased dramatically as a consequence of treatment with the anti-CSF1 Ab, compared to treatment with an isotype-matched control. There was a clear trend of decreased numbers also of CD68^+^ cells, although not significant (Figure 7A). CD115-immunostaining of liver sections visualized cells with myeloid morphology that decreased with anti-CSF1 Ab treatment (Figure 7B).

Importantly, CSF1 neutralization resulted in a significantly increased accumulation of endogenous HRG to levels around 300 µg/ml at day 7 of treatment with CSF1 Ab (Figure 7C) compared to 150 µg/ml in the control Ab treatment. The levels of plasma proteins fibrinogen and von Willebrand factor, analyzed at day 3 and 7 of treatment, were similar irrespective of whether mice had received the anti-CSF1 Ab or control Ab (data not shown). Liver *hrg* mRNA levels also remained unaffected (Figure 7D). Importantly, anti-CSF1 Ab-treated mice showed significantly reduced blood clearance of tail vein-injected ^125^I-mHRG at 15 min, but not 60 min, after injection (Figure 7E).

These data demonstrate that HRG uptake as well as turnover in blood is dependent on CSF1R-expressing mononuclear phagocytes.

## Discussion

In this study, we have extended the understanding of the regulation and distribution of HRG in healthy tissues and in tumors, essential for development of HRG-based therapeutics. Our data indicate that the turnover of HRG is quite rapid but due to the high rate of synthesis in the liver [29], the circulating levels remain intact. Analysis of human cancer showed that HRG was found in the circulation as expected but also associated with inflammatory cells in normal mucosa and CRC tissues. The specific binding of HRG to CD45^+^ inflammatory cells and the important role for CSF1R (CD115)-positive inflammatory cells in uptake and turnover of HRG that we report here, is in agreement with the finding that mononuclear phagocytes are key mediators of HRGs effects on tumor growth and metastasis [1]. Importantly, *hrg* gene targeting in mice profoundly affects peritoneal mononuclear cells, which are polarized towards a pro-angiogenic/immunosuppressive phenotype [16]. Identification of the exact phenotype of the HRG-binding monocyte/macrophage awaits characterization of the HRG receptor.

HRG turnover has been examined previously [30], leading to the conclusion that HRG has a half-life of several days in human plasma. This is in stark contrast to our results, showing a plasma half-life of less than 15 min for radiolabeled HRG. In the study by Lijnen et al., purified, plasma-derived human HRG was iodinated using the oxidation-dependent method of McFarlane [31], which is known to be considerably harsher than the Iodogene-mediated labeling used here, likely causing protein denaturation. Of note, both techniques result in modification of tyrosine and histidine residues. The histidine residues in the His/Pro domain are essential for HRGs bioactivity, e.g. by binding Zn^2+^ [4]. Excessive labeling would be detrimental to the folding and therefore function of HRG. We therefore took care to use mild conditions resulting in less than one iodine atom per HRG molecule, allowing the labeled HRG to maintain bioactivity as demonstrated by its ability to block chemotaxis of cells. We obtained the same results using purified, recombinant mouse and human HRG, and the results were compared to those for albumin, examined in parallel. The conditions in the current study were therefore optimized to draw conclusions relevant for the *in vivo* distribution and turnover of HRG.

Rabbit HRG cDNA was originally cloned by Borza and colleagues, who described the multidomain, disulfide-bonded molecular organization of HRG [8]. The different HRG domains may mediate different aspects of the multifaceted biology ascribed to HRG [4], [5], [7]. HRG bioactivity has been suggested by ourselves [17], and others [18], to involve HRG degradation. Based on HRG deletion mutants, we previously hypothesized that HRG needs to be fragmented to induce a biological effect, using endothelial chemotaxis as a surrogate assay [17]. Moreover, Poon *et al.* employed an *in vitro* system to show that plasmin-mediated cleavage of HRG may provide a feedback mechanism to regulate the effects of HRG on the plasminogen/plasmin system [18]. We analyzed CRC tissue for the presence of discrete HRG fragments using highly sensitive isoelectric focusing, which detected the same major full length HRG species in benign and CRC tissues, rather than fragments (Figure S6). While it is difficult to exclude the generation of bioactive HRG-derived fragments, our data point to that degradation of HRG is efficient and results in TCA-soluble peptides (i.e. <5 kDa; see Figure 2C, 2G), which were generated to an increased extent in tumor-bearing mice. This apparently complete degradation may occur as a result e.g. of uptake and lysosomal degradation in mononuclear cells or by extracellular proteolysis executed by plasmin or by matrix metalloproteinases, produced to an increased extent in the tumor microenvironment.

**Figure 8:**
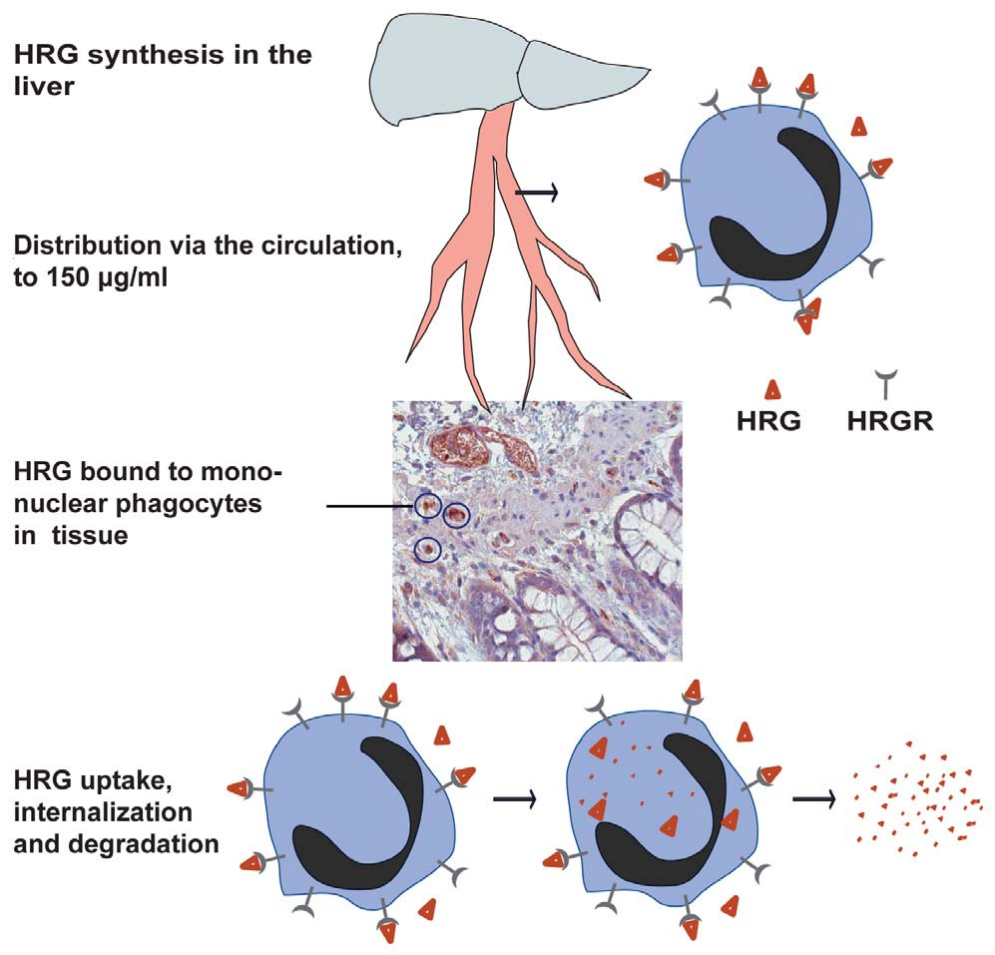
Schematic outline of HRG's interaction with its receptor on mononuclear phagocytes. HRG is shown produced in the liver, distributed in the circulation bound to mononuclear phagocytes which home to sites of inflammation. Binding of HRG to the HRG receptor (HRGR) leads internalization, degradation and thereby regulation of HRG turnover.

A number of novel data presented here support a model where mononuclear phagocytes express specific binding sites for HRG and are essential for HRGs distribution and biology: 1) HRG immunostaining was associated with inflammatory cells in normal and colorectal cancer tissue, 2) microautoradiography of tissues from T241-bearing mice injected with radiolabeled HRG showed colocalization of HRG with CD45^+^ cells, 3) radiolabeled HRG bound to specific, non-heparan sulfate dependent binding sites on a human mononuclear cell line, and 4) suppression of CSF1-dependent mononuclear phagocytes resulted in reduced uptake of radiolabeled HRG and accumulation of endogenous HRG in the blood. These data combined with our previous report that HRG induces gene regulation in peritoneal monocytes [16] make plausible the suggestion that HRG binds to a cell surface signaling receptor on mononuclear phagocytes (see Figure 8). The exact expression pattern of an HRG receptor awaits its molecular identification. This is a challenging but important task, which is required for the exploitation of HRG-based agonists in treatment of diseases characterized by inflammation and excessive angiogenesis.

## Conclusions

Administration of the heparin-binding plasma protein HRG to tumor-bearing mice has a decisive impact on the tumor microenvironment, leading to an improved vessel function and the mobilization of an anti-tumor immune response. The mechanism of action of HRG has remained unclear. Here, we provide new information on this abundant heparin-binding protein, its rapid turnover, unperturbed steady state levels, and its interaction with mononuclear cells *in vitro* and *in vivo*. Analyses are provided both of mouse models and human colorectal cancer tissues. For the first time, we identify direct, non-heparan sulfate-dependent binding to inflammatory cells, which we suggest mediate uptake of HRG from blood and turnover of HRG. Information on HRG's biodistribution and cellular target is a prerequisite for future development of HRG-based therapeutics.

## Supporting Information

Figure S1 **MicroPET/CT visualization of ^124^I-mHRG biodistribution in C57BL/6 mice.** Two individuals are shown. One animal (images 30 sec –20 min) was injected with ^124^I-mHRG through a tail-vein catheter followed by PET/CT acquisition. To obtain a snap-shot of radioactivity distribution at 1 h post injection, the mouse was euthanized, followed by PET/CT acquisition. See Methods S1 for details. Arrows indicate organs where radiolabeled HRG was enriched; H; heart, K; kidney, L; liver, S; spleen, T; thyroid.
(TIF)

Figure S2 **mHRG plasma levels.** The levels of endogenous mHRG was measured using ELISA, in mice with Panc02 pancreatic cancer compared to naive control mice.
(TIF)

Figure S3 **Mouse liver HRG transcripts in livers from naive and tumor-bearing mice.** Livers were harvested from naive control mice or mice challenged with T241 fibrosarcoma for 11 days (early) or 21 days (late). Control n = 10, T241 day 11 (early) n = 4, T241 day 21 (late) n = 5. *, p<0.05; Student's t-test.
(TIF)

Figure S4 **Scoring of CD45-specific IHC signals in CRC arrays.** The number, n, of biopsies were; normal = 10, adenoma = 10, stage 1 = 20, stage 2 = 20, stage 3 = 17, distant metastasis = 20.
(TIF)

Figure S5 **Uptake of 555-HRG in the RAW264.7 macrophage cell line.** Incubation of RAW264.7 cells with 555-labeled HRG shown by fluorescence microscopy (left) and light microscopy (right). Staining with DAPI (blue) shows nuclei.
(TIF)

Figure S6 **Isoelectric focusing using NanoPro of HRG in colorectal cancer tissue.** A. Electropherogram from NanoPro isoelectric focusing, showing two peaks, P1 and P2, detected using the anti-His-Pro domain antibody in a typical CRC biopsy. B. Quantification of P1 in biopsies from healthy individuals or individuals with benign polyps (n = 17), stage 2 CRC (n = 16) and stage 4 CRC (n = 16). The P1 peak area for each individual sample was determined and normalized to HSP-70. C. Quantification of P2 in biopsies from healthy individuals or individuals with benign polyps (n = 17), stage 2 CRC (n = 16) and stage 4 CRC (n = 16). The P2 peak area for each individual sample was determined and normalized to HSP-70.
(TIF)

Methods S1 (DOCX)
