# Correction: Histidine-Rich Glycoprotein Uptake and Turnover Is Mediated by Mononuclear Phagocytes

**DOI:** 10.1371/journal.pone.0118636

**Published:** 2015-03-05

**Authors:** The PLOS ONE Staff

Fig. 2 is incorrect. The y-axis is incorrectly labeled in Fig. 2G. The authors have provided a corrected version here.

**Fig 2:**
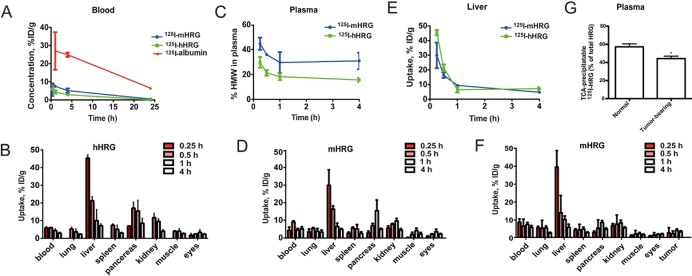
Unusually rapid biodistribution of radiolabeled HRG. A. Blood kinetics of ^125^l-albumin, ^125^l-hHRG and ^125^l-mHRG in C57BL/6 mice. (n = 4/time point). B. Biodistribution of ^125^l-hHRG in selected C57BL/6 mouse organs. C. Percentage of radioactivity in blood plasma, associated with a high molecular-weight fraction (>5 kDa). D. Biodistribution of ^125^l-mHRG in selected organs of naive C57BL/6 mice. E. Liver uptake of ^125^l-mHRG and ^125^l-mHRG. F. Biodistribution of ^125^l-mHRG in selected organs of T241 fibrosarcoma-bearing C57BL/6 mice. G. TCA-precipitable ^125^l-radioactivity in plasma after 2 h of circulation in naive and tumor-bearing mice injected with ^125^l-mHRG.

The Fig. 4 legend is incorrect. The third sentence of the Fig. 4 legend should have been omitted. The complete, correct Fig. 4 legend is:

**Fig 4:**
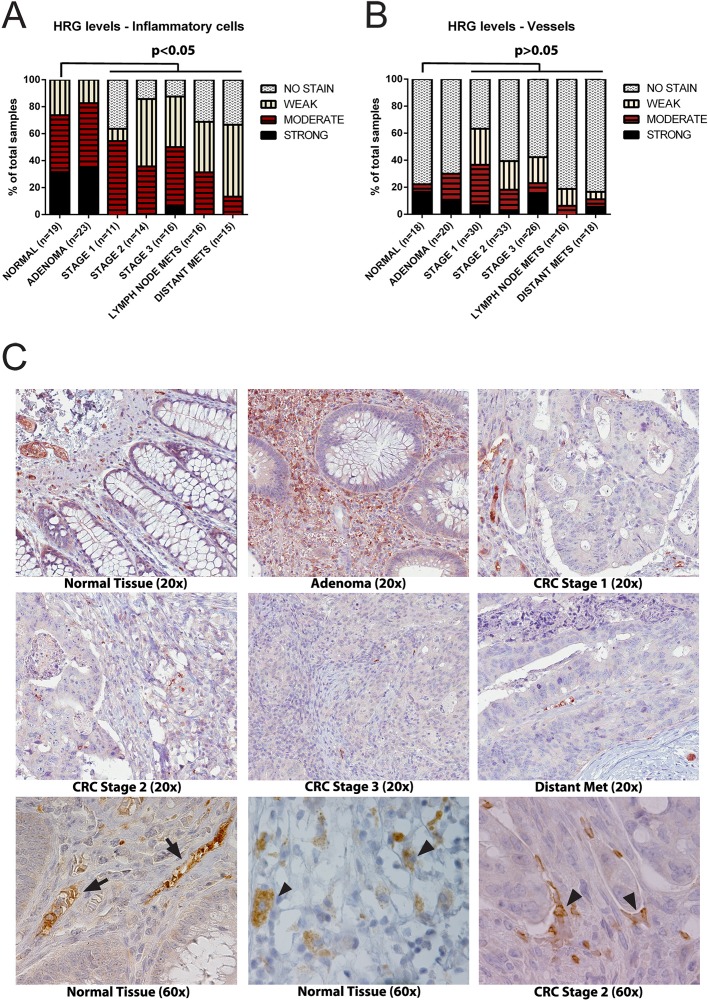
HRG detected by IHC of CRC tumor tissue arrays. A. Scoring of HRG IHC signals associated with inflammatory cells in CRC arrays from strong to no signal. Statistical analysis; p<0.05 was considered significant. B. Scoring of HRG IHC signals associated with vessels, as above. C. Upper and middle row of panels: Representative images of the HRG IHC signals from the indicated categories at 20× magnification. Lower row of panels: Representative images of the HRC IHC signals in CRC at 60× magnification. Arrows indicate typical vessel-associated HRG signals in normal colorectal tissue (left) and in inflammatory cells in normal tissue (middle) and in stage 2 CRC (right).
