# Supplementary figures and images for: Histidine-Rich Glycoprotein Uptake and Turnover Is Mediated by Mononuclear Phagocytes
Source: PLoS One. 2014 Sep 22;9(9):e107483. doi: 10.1371/journal.pone.0107483 (PMC4171488; doi:10.1371/journal.pone.0107483)

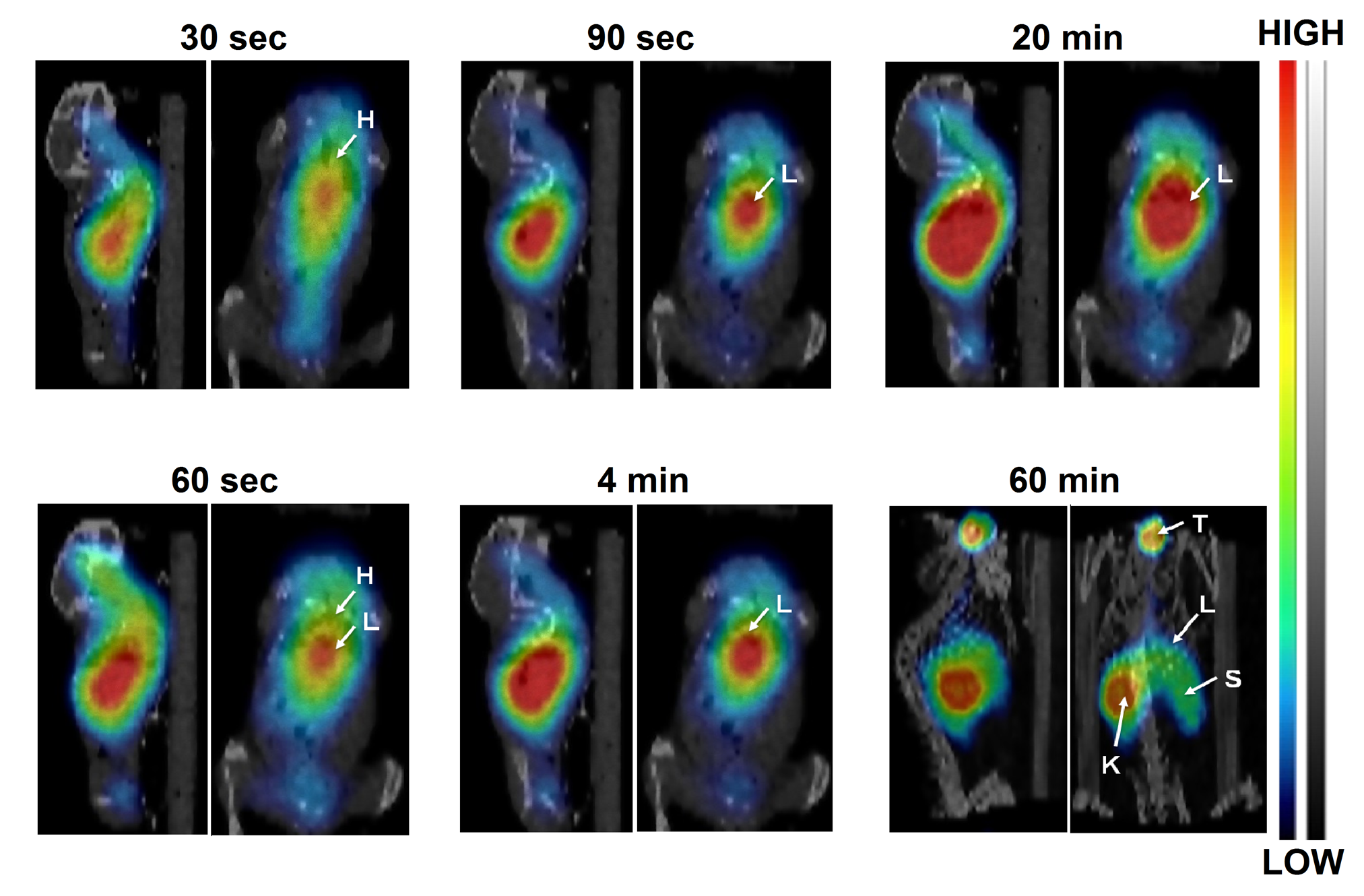

Supplement: Figure S1 — MicroPET/CT visualization of 124I-mHRG biodistribution in C57BL/6 mice. Two individuals are shown. One animal (images 30 sec –20 min) was injected with 124I-mHRG through a tail-vein catheter followed by PET/CT acquisition. To obtain a snap-shot of radioactivity distribution at 1 h post injection, the mouse was euthanized, followed by PET/CT acquisition. See Methods S1 for details. Arrows indicate organs where radiolabeled HRG was enriched; H; heart, K; kidney, L; liver, S; spleen, T; thyroid. (TIF) [file pone.0107483.s001.tif]

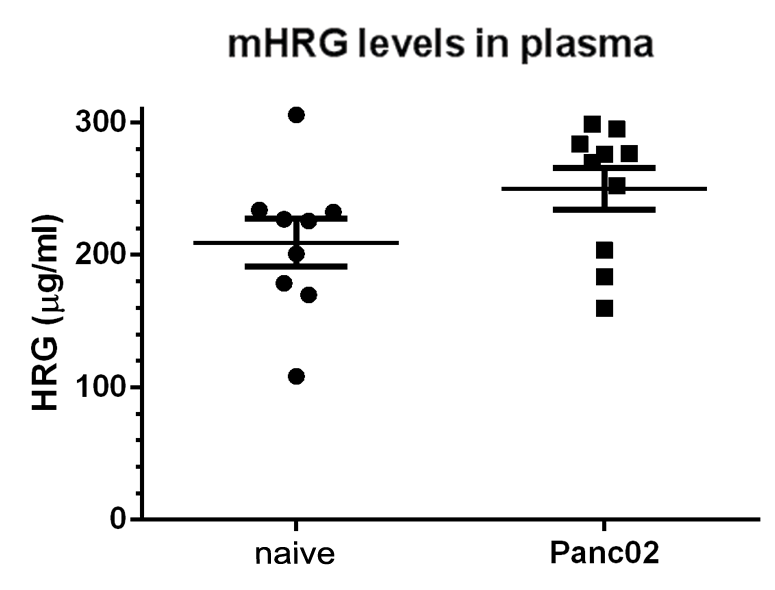

Supplement: Figure S2 — mHRG plasma levels. The levels of endogenous mHRG was measured using ELISA, in mice with Panc02 pancreatic cancer compared to naive control mice. (TIF) [file pone.0107483.s002.tif]

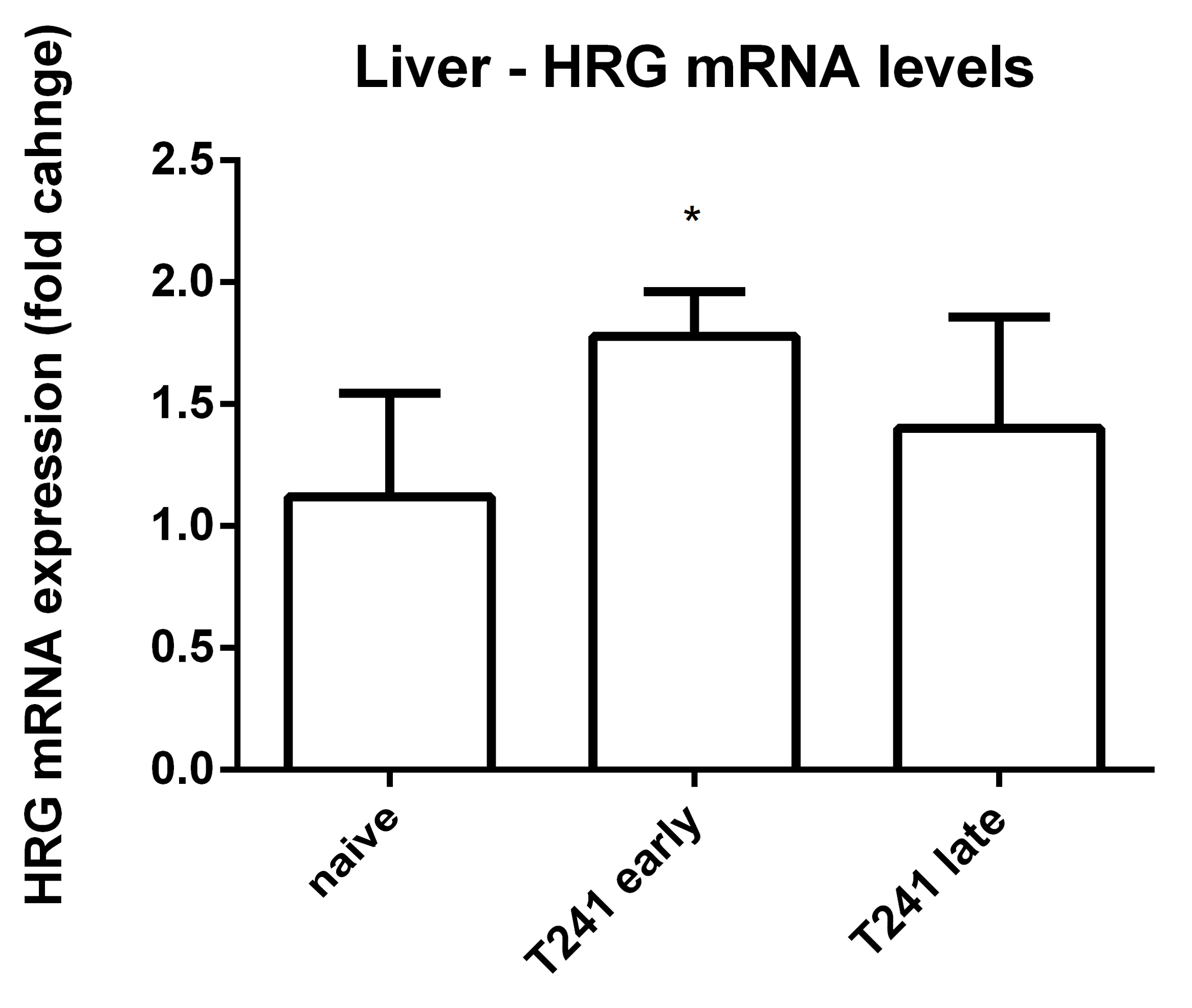

Supplement: Figure S3 — Mouse liver HRG transcripts in livers from naive and tumor-bearing mice. Livers were harvested from naive control mice or mice challenged with T241 fibrosarcoma for 11 days (early) or 21 days (late). Control n = 10, T241 day 11 (early) n = 4, T241 day 21 (late) n = 5. *, p<0.05; Student's t-test. (TIF) [file pone.0107483.s003.tif]

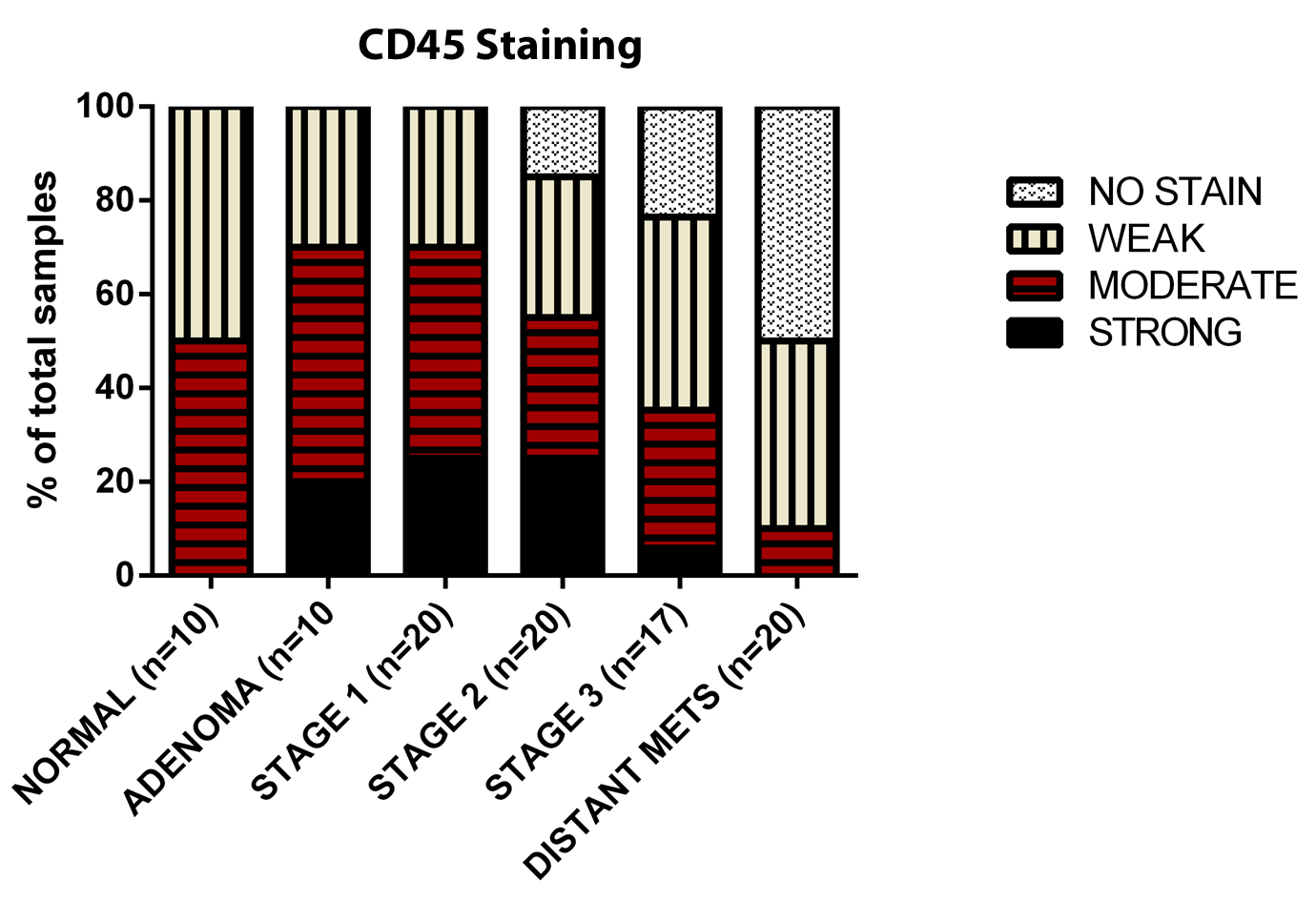

Supplement: Figure S4 — Scoring of CD45-specific IHC signals in CRC arrays. The number, n, of biopsies were; normal = 10, adenoma = 10, stage 1 = 20, stage 2 = 20, stage 3 = 17, distant metastasis = 20. (TIF) [file pone.0107483.s004.tif]

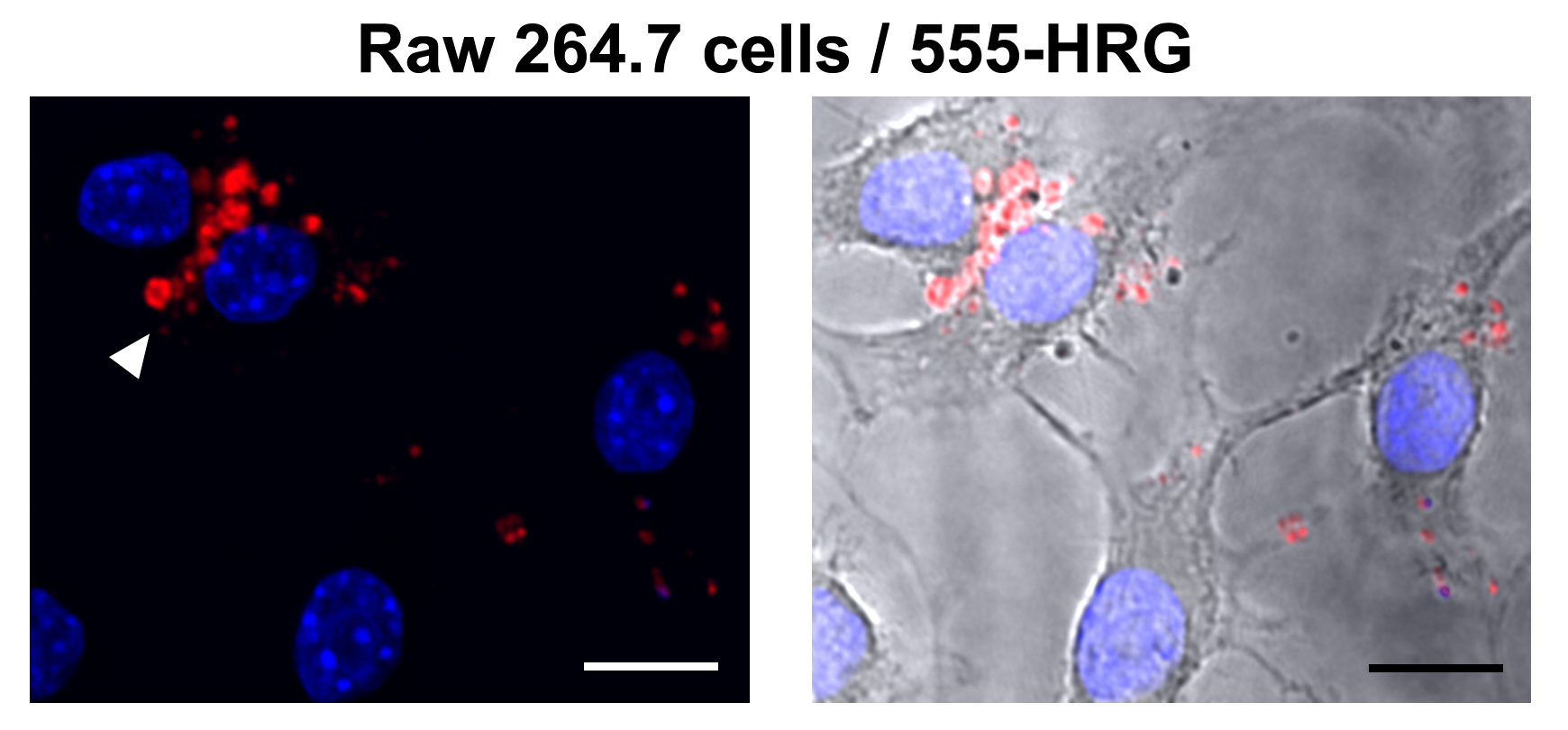

Supplement: Figure S5 — Uptake of 555-HRG in the RAW264.7 macrophage cell line. Incubation of RAW264.7 cells with 555-labeled HRG shown by fluorescence microscopy (left) and light microscopy (right). Staining with DAPI (blue) shows nuclei. (TIF) [file pone.0107483.s005.tif]

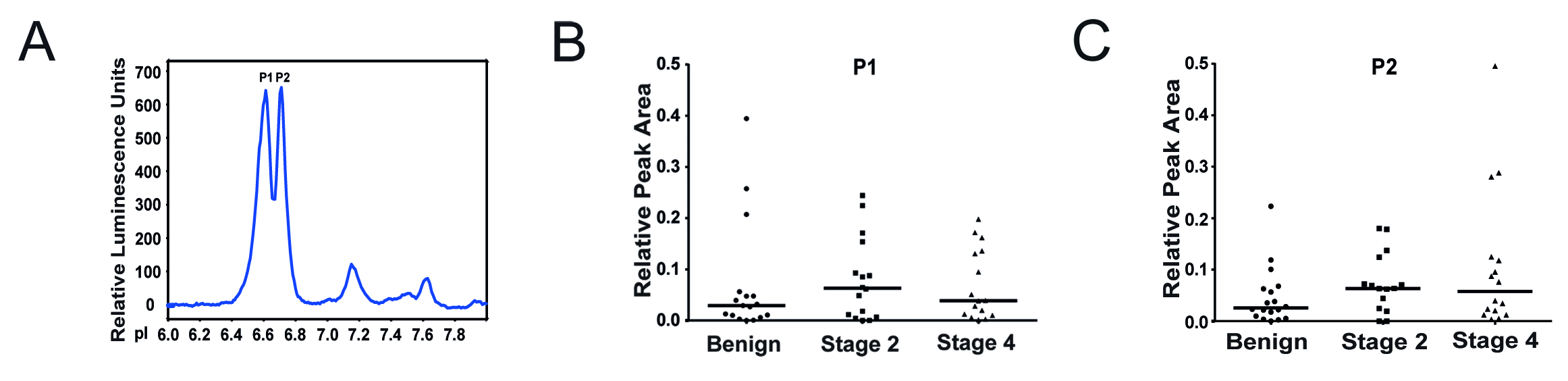

Supplement: Figure S6 — Isoelectric focusing using NanoPro of HRG in colorectal cancer tissue. A. Electropherogram from NanoPro isoelectric focusing, showing two peaks, P1 and P2, detected using the anti-His-Pro domain antibody in a typical CRC biopsy. B. Quantification of P1 in biopsies from healthy individuals or individuals with benign polyps (n = 17), stage 2 CRC (n = 16) and stage 4 CRC (n = 16). The P1 peak area for each individual sample was determined and normalized to HSP-70. C. Quantification of P2 in biopsies from healthy individuals or individuals with benign polyps (n = 17), stage 2 CRC (n = 16) and stage 4 CRC (n = 16). The P2 peak area for each individual sample was determined and normalized to HSP-70. (TIF) [file pone.0107483.s006.tif]
