# Supplementary material for: Histidine-Rich Glycoprotein Uptake and Turnover Is Mediated by Mononuclear Phagocytes
Source: PLoS One. 2014 Sep 22;9(9):e107483. doi: 10.1371/journal.pone.0107483 (PMC4171488; doi:10.1371/journal.pone.0107483)
Supplement: Methods S1 — (DOCX) [file pone.0107483.s007.docx]

**Supplemental Methods S1**

**Biodistribution of ^124^I-mHRG using microPET:** To obtain visual confirmation of HRGs biodistribution, a microPET/CT(Triumph™Trimodality System, TriFoil Imaging, Inc., Northridge, CA, USA) study was performed on C57BL/6 mice after injection of 2 MBq/5 µg of ^124^I-mHRG in 100 µl PBS. Because C57BL/6 tolerate isoflurane anesthesia poorly, the imaging was performed in two sessions. One animal was catheterized in the tail vein under anesthesia, and placed on a warm pad. The animal was then injected i.v. with ^124^I-mHRG through the catheter. A PET acquisition was performed in the list mode, up to 20 min post injection. CT acquisition was performed using field of view 80 mm, magnification 1.48, one projection and 512 frames for 2.13 min. To obtain a snap-shot of radioactivity distribution at 1 h post injection, an injected mouse was sedated with CO_2_ and sacrificed by cervical dislocation. A cadaver was placed in the camera, and after a CT scan, a PET acquisition was performed for 30 min. The PET data were reconstructed as images using a MLEM 2D algorithm (10 iterations). The CT raw files were reconstructed using Filter Back Projection (FBP). µPET and CT data as DICOM files were analyzed using PMOD v3.13 (PMOD Technologies Ltd, ZRH, Switzerland).

**Instrumentation:** Radioactivity was measured using an automated gamma-counter with a 3-inch NaI(Tl) detector (1480 WIZARD, Wallac Oy). Distribution of radioactivity along thin layer chromatography strips and PAGE gels was measured on a Cyclone^TM^ Storage Phosphor System and analyzed using the OptiQuant^TM^ image analysis software. For formulation of injection solution, radioactivity was measured using a dose calibrator VDC-405 (Veenstra Instruments BV, Holland) equipped with an ionization chamber. The microSPECT/CT study was performed using the Triumph™ Trimodality system (Gamma Medica, Inc.), a fully integrated SPECT/PET/CT hardware and software platform optimized for small animals in pre-clinical applications.

**Orthotopic pancreas cancer study:** Orthotopic pancreatic tumor challenge was performed by injection of Panc02 cells in mice anesthetized with isoflurane. The stomach was exteriorized via abdominal midline incision, and 10^6^ Panc02 tumor cells in 30 ml PBS were injected. Plasma and tumors were collected 11 days after injection.

**HRG fluorescent labeling and immunolocalization:** Alexa-555 (A555) was coupled to HRG (60 µg) using an Alexa Fluor 555 Microscale Protein Labeling kit (Invitrogen) according to manufacturer’s instructions. RAW 264.7 murine macrophages (75,000 cells per cm^2^) were seeded on coverslips in 24-well plates and incubated over night at 37°C. Cells were incubated with 5-10 µg/ml of 555-HRG for 1 h and then fixed for 10 minutes at room temperature with 4% PFA. Coverslips were mounted with Fluoromont mounting medium on microscope slides. Samples were analyzed using a LSM700 MetaConfocal microscope (CarlZeiss) and pictures taken at 63 x magnifications with an oil immersion objective.

**NanoPro isoelectric focusing:** Human CRC biopsies were lysed in RIPA buffer containing phosphatase and protease inhibitors (ProteinSimple, Santa Clara, CA). Lysates were clarified by centrifugation and protein concentration determined using the BCA Protein Assay Kit (Pierce, Rockford, IL, USA). Lysates, run in triplicate, were mixed with ampholyte premix (pH 3-10) and fluorescent pI standards (pI Standard Ladder 3) before loading into the NanoPro1000 system (ProteinSimple). Isoelectric focusing was performed in capillaries filled with a mixture of cell lysate (0.05-0.1 mg/ml protein), fluorescently labeled pI standards, and ampholytes. The separated proteins were cross-linked to the capillary wall using UV light followed by immunoprobing with rabbit 0119 anti-HRG antibodies raised against the His/Pro domain [17]. HSP-70 antibodies were run in parallel for normalization. HRPconjugated secondary antibodies (anti rabbit-HRP; ProteinSimple) were used to detect the signal. The signal was visualized by ECL and was captured by a CCD camera. The digital image was analyzed and peak area quantified with Compass software (ProteinSimple).
